# Cold Water Cure of Inebriety

**Published:** 1911-08

**Authors:** T. D. Crothers

**Affiliations:** Superintendent Walnut Lodge Hospital, Hartford, Connecticut


THE
### TEXAS MEDICAL JOURNAL.
Established July, 1885

F. E. DANIEL, M. D., - - - - - - Editor, Publisher and Proprietor

Published Monthly.—Subscription $1.00 a Year

VOL. XXVII. AUSTIN, AUGUST, 1911. No. 2.

The publisher is not responsible for the views of the contributors.

### Original Articles.

For Texas Medical Journal.

### Cold Water Cure of Inebriety.

BY T. D. CROTHERS, M. D., SUPERINTENDENT WALNUT LODGE HOSPITAL, HARTFORD, CONNECTICUT.

The weird story of Dr. Bruno,* with its subtle psychological possibilities and intimations of great unknown laws and forces yet to be discovered, suggests the story of Rev. Dr. Brown.

There were some psychological aspects in his case which suggest Dr. Bruno's theories, only on a more simple plan. This is one of the very curious cases that has come to my notice during thirty years of constant association with border-liners who use spirits and drugs.

Rev. Dr. Brown was one of the most popular and fascinating clergymen in a large Eastern city. He had an immense church and a great following. He was married and had a family nearly grown up which was greatly devoted to him. His life was a very strenuous one. Sunday and week-day services, besides lectures, after dinner speeches and interviews with the press, kept up a continual whirl of excitement.

One of his devoted parishioners, a physician, consulted me about Dr. Brown's secret use of spirits. For a long time he suffered from insomnia, and spirits were found to be a pleasing anesthetic. This continued until almost every night he was obliged to use some form of alcohol. Various remedies were tried without relief.

*"The Strange Case of Dr. Bruno," F. E. Daniel, M. D. Von Boeckmann-Jones Co., Austin, Texas. $1.50 postpaid.

In a consultation he told me that when eighteen years of age a gypsy fortune teller had told him that he "would break down and go into a black cloud at forty-eight years of age, and this time had now arrived, and he was convinced that the prediction was true, and that the use of alcohol could not be checked. He would die if he did not have it, and this was the cloud into which he was drifting rapidly.

His grandfather at fifty years of age suddenly became an inebriate, and later became insane and died a few years afterward. He had a conviction which he could not throw off that he would follow in his footsteps. This obsession he was not able to overcome, and while he reasoned out its absurdity and sought in every way to drive the thought from his mind, it remained.

In the morning, after a night of heavy drinking, the depression was so great as to suggest suicide. This condition went on for a year or more. It was finally suggested that in company with the family physician he go on a yacht excursion along the coast. Notwithstanding every effort on the part of the physician, he secreted several bottles of brandy, and the first night out was greatly intoxicated. The second night his condition increased, and with it an intense depression and low grade of delirium.

The physician resolved to try the experiment of shock. One day while staggering on the deck a rope was tied around his body and he was thrown overboard. The shock and strangulation from swallowing large quantities of salt water sobered him in part. and the doctor realizing the safety of the experiment, allowed him to remain in the water for some little time, then with a great show of excitement and alarm pulled him in, and accused him of trying to commit suicide.

The clergyman was greatly horrified, and every effort was made to deepen his fear and impress on him the danger of collapse. The horror of drink had settled on his mind, and he was taken home and treated with the usual tonics, followed by recovery.

His hair turned gray and his usual buoyancy of manner was subdued and changed. Although cheerful, there was a reserve and quiet dignity unknown before. A few months later I was sent for in great haste to consult with his physician. He had suddenly been overtaken with a desire to drink and had gratified it, and on awakening the desire was so intense and the horror to overcome it brought on a high fever.

In view of the effect of the ocean bath it was decided to try something similar. A tub of cold water was drawn and he was forcibly taken to it and several pails of water were dashed over his head. He was taken out and vigorously rubbed and returned to the bath with the same experience.

He did not offer any resistance, but suddenly he exclaimed, "I am cured. The desire has passed away. I shall never drink again." This proved to be true. While weak and timid, his former intellectual ability reappeared. Suddenly he became a very pronounced temperance man, affirming that he had a revelation and that henceforth he must work for the inebriate. He carried his church audience with more than his old time vigor and popularity.

A year later a severe rheumatic fever came on. The doctor treated him as before, with cold showers and forcible douches, followed by vigorous rubbing, and he recovered quickly.

The former obsession that he was to go under a cloud disappeared, and in its place a burning zeal to have every one sign the pledge and become total abstainers. His pulpit work retained its same brilliancy of intellectual effort, but outside of that he was a burning, consuming temperance advocate, who sought every opportunity and occasion to preach temperance and health in its broadest aspect.

He consulted physicians on health matters and sought all the possible information he could concerning the causes of the drink evil. At night he would go down to saloons and plead with barkeepers and saloon men to give up the business. His pulpit work contained little or no reference to his sanitary teachings.

Among his friends he was regarded as eccentric and partially insane on this subject. This continued for several years without much change. Out of the pulpit, on the lecture platform, and among his friends he was the great apostle of temperate living. Privately he had distinct attacks of depressive melancholia, in which the fear of drinking and suicide produced intense fever. This was treated with cold baths and showers, as before, with the best results.

Finally he confided to the physician that he would die on his sixty-first birthday at 10 o'clock in the morning, also that he would send in his resignation of his pastorage the week before, and have it read before a church meeting on the day he would die.

His physician treated this as a delusion and did everything to break up the impression. In consultation with me he said with great calmness that this was inevitable, although there was no evidence of it. No reasoning could break up the idea.

He continued his work with all the usual signs of vigor and excellence, and this secret obsession was only known to his wife and physician. Nothing was said about it. The doctor in the meantime watched him narrowly for signs of physical depression, giving him a vigorous tonic treatment, which seemed to overcome all shades of depression and place him in the best possible health.

On the morning of the sixty-first birthday he awoke in his usual health, met the doctor, who called early, and arranged for several events that were to occur during the week. He was cheerful, wrote a note declining an invitation to attend a meeting the next day, and conversed about various things with the doctor.

Finally an old clock on the stairs struck the hour of ten. He arose quietly, laid down on the lounge, drew one or two long breaths and died. The heart seemed to have stopped at once. There was no excitement, no utterance, none of the usual death-bed scenes, but sudden and profound collapse.

His wife in a nearby room had no conception of the reality, and supposed his statements were only the impulses of the moment growing out of some depressed state.

Evidently the water and the doctor's wise application of it in the form of a shock broke up the obsession that he was going into a "great cloud" in middle life.

Where the other obsession came from, it was impossible to say. He was not a visionary man, but optimistic and hopeful; one who loved life and his family and had no thought of death, or had never intimated it to any of his friends except the doctor and his wife, that the end would come at a certain time.

The doctor, a skillful diagnostician and a practical psychologist, considered this a psychical death at which time the organism stopped at a certain point, determined by some unknown power before.

Repeated examinations some months before his death indicated a high degree of health and a large life insurance policy was considered a good risk.

It was evident that the water had broken up the drink craze for the time being and diverted the morbid energies into other paths. Whether a shock in the form of water at the last moment might have everted the collapse, it is difficult to say.

The physician was prepared to resort to this agent again if there was any evidence of his usual feverish state or any abnormal condition, but in the absence of any indications he, too, was startled by the sudden fulfillment of the prediction.

Verily, Dr. Bruno's "strange case" is a border-land record that might be duplicated in some particulars in the experience of many physicians.

